# Prevalence, associated factors and health impact of intimate partner violence against women in different life stages

**DOI:** 10.1371/journal.pone.0221049

**Published:** 2019-10-09

**Authors:** Belén Sanz-Barbero, Natalia Barón, Carmen Vives-Cases

**Affiliations:** 1 Department of Epidemiology and Biostatics. National School of Public Health, Institute of Health Carlos III. Madrid, Spain; 2 Consortium for Biomedical Research in Epidemiology & Public Health (CIBERESP), Madrid, Spain; 3 Joint Research Institute National Distance Education University and Institute of Health Carlos III (IMIENS), Madrid, Spain; 4 Public Health Research Group, Department of Community Nursing, Preventive Medicine and Public Health and History of Science, Alicante University, Alicante, Spain; University of Westminster, UNITED KINGDOM

## Abstract

**Objectives:**

The effect of age on intimate partner violence (IPV) against women has received little attention. The objective of this study is to analyze the prevalence, risk factors and health impact of current IPV in different life stages.

**Methods:**

We analyzed a sub-sample of 8,935 ever-partnered women aged 16 years and older from the Spanish Macrosurvey on Gender Violence of 2014. Main outcomes: current physical/ sexual IPV and current psychological-only IPV. The impact of IPV on health was analyzed using the variables self-perceived health, mental health and activity limitations. Risk factors were assessed using the prevalence ratio (PR) from Poisson regression models with robust variance. Analyses were stratified by age (young people, adults, and elderly people).

**Results:**

Abuse in childhood increases the likelihood of IPV in any life stage. A higher education level decreases the probability of physical/sexual IPV across all ages. Unemployment increases the probability of IPV in adult women (physical/sexual-IPV, PR:1.7; psychological-IPV, PR:1.3). Being an immigrant increases the likelihood of physical/sexual IPV in adult women (PRwomen:1.91). Women exposed to current physical/sexual IPV have a greater likelihood of reporting poor self-perceived health (PRyoungpeople:2.59; PRadults:1.68; PRelderly:1.28), poor mental health (PRyoungpeople:3.10; PRadults:2.61; PRedlerly:2.17) and activity limitations (PRyoungpeople:2.44; PRadults:1.98). For psychological IPV only, there is an increase in the probability of poor self-perceived health (PRadults:1.37; PRelderly:1.19), poor mental health (PRyoungpeople:2.24; PRadults:2.16; PRelderly:1.69), and activity limitations (PRadults:1.30; PRelderly:1.18).

**Conclusions:**

We found both common factors and differential factors when looking at IPV by age group. This shows the need to link gender violence prevention with the social circumstances of the population across different life stages.

## Introduction

Gender violence (GV) is an important public health problem and is internationally recognized as a serious violation of women’s human rights. [1] In some contexts, addressing GV has become a government priority due to the social pressure to address a problem that has serious consequences for the health and wellbeing of women and families. In addition, it generates high social and economic costs for the whole population. [2]

IPV is one of the forms of GV that has been most documented in epidemiological literature to date. It is estimated that 47% of world femicides are carried out by a woman’s intimate partner or by a family member [3] and that 30% of women worldwide have suffered some sort of physical and/or sexual IPV. [4] In the European context, one in five women has been a victim of physical and/or sexual IPV. In Spain, the prevalence is around 13% and reaches 33% among women who suffer psychological IPV previously. [5] It is precisely due to the fact that this type of violence is produced within intimate relationships that IPV has such a strong impact on women’s identity, above all on their self-esteem and health. It can cause psychological damage that remains throughout a woman’s entire life. [6]

The majority of the epidemiological studies that analyze the causes and consequences of IPV have been carried out among the general population, [6,7] however, there is increasing scientific evidence that shows an increase in IPV among the young population. [8] A recently published multi-country study (carried out with a representative sample of 28 countries in the European Union) shows a current prevalence of physical and/or sexual IPV in women ages 18–29 of 6.1% (4% among the general population) and a lifetime prevalence of psychological IPV of up to 47.9% (32% among the general population). [5] Also, being in elder ages has been identified as a barrier to leaving a violent relationship. [9,10] It is possible that age interacts with conditions such as immigrant status, social support, economic dependence, functional dependence, etc., and that this intensifies women’s vulnerability to IPV in different ways across the stages of life. [11]

The objective of this study is to analyze the prevalence, associated factors and health impact of current IPV among women in different life stages. The analysis of current IPV in different stages of life can provide useful information to support the development of strategies that address IPV according to the specific needs of groups of women of different ages.

## Methods

### Study population

This is a cross-sectional study of the “Macrosurvey of Violence Against Women” carried out in 2014 in a sample of 10,171 women residing in Spain ages 16 and older. [12] The survey included a multi-stage sampling process, stratified by conglomerates, with the selection of primary sample units (municipalities) and secondary units (sections) by proportional randomization. The final units (individuals) were selected by random routes and quotas based on age and occupation. Information was collected through personal interviews in people’s homes. The database “Macro survey of Violence against Women” is open and public. The records are anonymous. Microdata can be requested at the following link: http://www.cis.es/cis/opencms/ES/formulario.jsp?dwld=/Microdatos/MD3027.zip

This study analyzes a sub-sample that corresponds to women who have and have had a partner at some point in their life (n = 9,805 women). Given the nature of our independent variable (exposure to IPV in the past 12 months), we excluded women who reported having a single partner over the course of their lives and who had died at least one year prior (n = 870 women). So, we analyzed a sub-sample of 8,935 ever-partnered women.

### Measurements

The dependent variables analyzed refer to physical, sexual and/or psychological violence against women, in the past 12 months, in the context of a current or prior intimate relationship. Following the “Guidelines for the production of statistics on violence against women: statistical surveys” published by the United Nations [13] we considered a woman to have suffered severe physical violence when she reported that her partner had “tried to smother or burn her intentionally” or had “threatened her with a gun, knife or other weapon”. A woman was considered to have been exposed to moderate physical violence when she reported aggressions such as being punched, hit, kick, dragged, etc. Sexual violence includes aggressions such as the obligation or intent to have sex against her will, the acceptance of sex due to fear, as well as receiving humiliating treatment against her will. Psychological violence includes: psychological control and attitudes related to jealousy, monitoring of women’s agendas and activities and the imposition of limitations on who women can relate to among friends and family members. Emotional violence includes attitudes related to humiliation, insult, devaluing, and verbal threats and intimidation, and economic violence refers to control of domestic finances and the decrease in women’s economic independence through prohibition of a woman’s pursuit of work or studies outside of the home.

The impact of IPV on health was analyzed through the following dependent variables:

Perceived health, recoded as good (good, very good) and poor (regular, poor and very poor); mental health: based on a list of eight symptoms that include wanting to cry for no reason, changes in emotional state, anxiety or anguish, low sex drive, irritability, insomnia, permanent fatigue, and sadness due to feelings of worthlessness. The variable was recoded into presence of 0–3 symptoms and 4–8 symptoms. Activity limitations: presence/absence of health problems that limit or can be foreseen to limit daily activities for one year or more.

Based on prior studies, [14–17] the covariables analyzed using logistical regression models include country of birth (Spain/ foreigner), education level (primary/ secondary/ higher education), employment situation (paid worker/ unemployed/other), social support (almost always or always/ sometimes, never, or almost never) and exposure to physical and/or sexual violence prior to age 15 by an adult (yes/ no).

Analyses were carried out with stratification by three age groups: young women (ages 16 to 29), adult women (ages 30 to 49) and elderly women (ages 50 and over).

### Statistical analysis

Prevalence and the prevalence ratio (PR) was calculated with 95% CI for the different types of IPV to which women have been exposed on the past 12 months, using the young population as the reference group for the calculation of PR.

The association measure used to analyze the variables associated with physical and/or sexual IPV, and psychological IPV only, as well as the variables related to state of health was PR, analyzed using Poisson regression models, with robust variance. A univariate analysis and later multivariate analysis were carried out. In order to avoid biases due to erroneous classification, the regression analysis considered women exposed to IPV in comparison to women who had never suffered any type of violence.

Prior to the inclusion of the covariables in the models, we analyzed the correlation between the different covariables using Spearman’s correlation coefficient, with a maximum accepted correlation coefficient value of 0.45. [17] Throughout the analysis we used weights included in the Gender Violence Macrosurvey. Calculations were carried out using the statistical software SPSS 21.0 and STATA 14.

## Results

Around 15.6% of women included in our sample had been exposed to some type of IPV during the past 12 months. Specifically, 12.2% of women suffered only psychological IPV and 3.1% was exposed to physical and/or sexual IPV.

Table 1 shows the prevalence of different types of IPV by age group in the whole sample as well as the prevalence ratios using young women ages 16 to 29 as the reference group. For all types of IPV analyzed, the prevalence of IPV is greater among young women than among adult women ages 30–49 and those over age 50. Using adult women as the reference group results in the prevalence of physical IPV decreasing by 38% in adult women [PR (IC95%): 0.62 (0.44; 0.86)] and 69% in elderly women [PR (IC95%): 0.31 (0.21; 0.46); sexual IPV prevalence decreases by 48% in elderly women [PR (IC95%): 0.52 (0.33; 0.82)]; the prevalence of psychological IPV decreases by 32% in adult women [PR (IC95%): 0.68 (0.60; 0.77) and by 41% by elderly women [PR (IC95%): 0.59 (0.51; 0.67) compared to young women.

**Table 1:** Prevalence and prevalence rates of different types of inter-partner violence among women exposed in the last 12 months, in the entire simple and by age group.

	Young people		Adults				Elderly				Total	
Typology of inter-partner violence	16–29 years (n = 1528)		30–49 years (n = 3718)				50 years and over (n = 3689)				(n = 8935)	
	%	RP	%	RP	IC 95%		%	RP	IC 95%		n	%
Physical IPV (nc = 120)	(3.8)	1	(2.4)	0.62	(0.44	0.86)	(1.2)	0.31	(0.21	0.46)	184	(2.1)
Physical-moderate	(1.9)	1	(1.1)	0.56	(0.35	0.90)	(0.6)	0.28	(0.16	0.50)	90	(1.0)
Physical severe	-	-	-	-	-	-	-	-	-	-	11	( - )
Both	(1.7)	1	(1.1)	0.63	(0.38	1.04)	(0.6)	0.32	(0.18	0.59)	85	(1.0)
Sexual IPV (nc = 105)	(2.3)	1	(1.8)	0.75	(0.49	1.14)	(1.2)	0.52	(0.33	0.82)	142	(1.7)
Psychological IPV (nc = 278)	(21.7)	1	(14.7)	0.68	(0.60	0.77)	(12.8)	0.59	(0.51	0.67)	1318	(15.2)
Psychological control-related (nc = 191)	(18.4)	1	(9.8)	0.53	(0.46	0.62)	(8.2)	0.45	(0.38	0.52)	940	(10.7)
Psychological economic (nc = 219)	(3.0)	1	(3.5)	1.19	(0.85	1.68)	(2.3)	0.78	(0.54	1.13)	260	(2.9)
Psychological emotional (nc = 128)	(10.7)	1	(9.2)	0.85	(0.71	1.02)	(8.3)	0.77	(0.64	0.93)	796	(9.1)

n: unweighted frequency; % weighted percentage; IPV: Intimate partner violence

**Fig 1:**
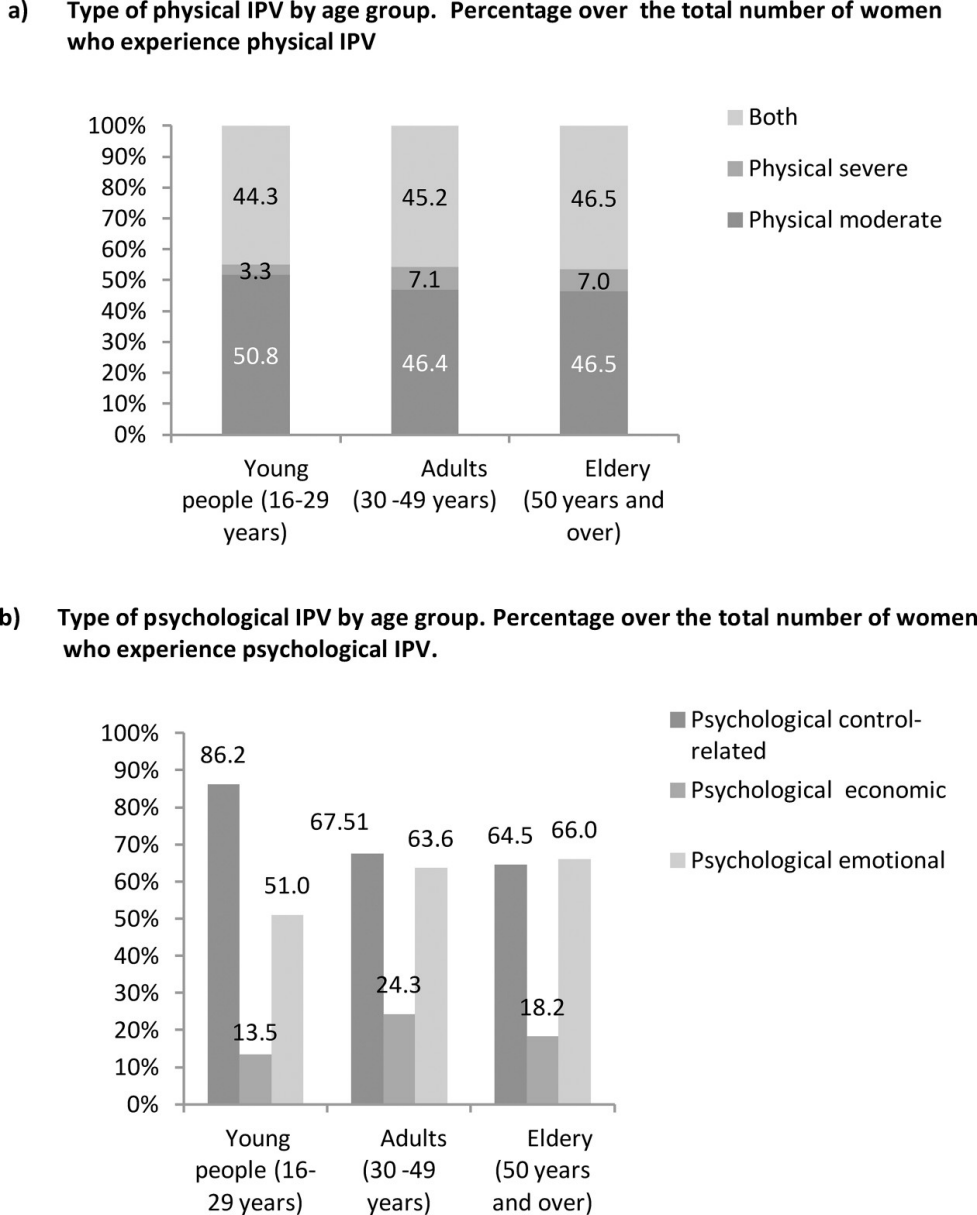
Type of current physical IPV (1a) and current psychological (1b) IPV by age group. a)Type of physical IPV by age group. Percentage over the total number of women who experience physical IPV b)Type of psychological IPV by age group. Percentage over the total number of women who experience psychological IPV.

The type of IPV shows differences by age group. Based on the total number of women who have suffered current physical IPV (Fig 1A), the percentage of women that report moderate physical IPV (without severe violence) is greater in young women than in adult and elderly women (50.8% *vs* 46.4% *vs* 46.5%), while the percentage of women who report both types of physical IPV (moderate and severe) is greater in elderly women (46.5%) than in young women (44.3%). In relation to current psychological IPV (Fig 1B), 86.2% of young women that have suffered from psychological IPV report control-based psychological IPV, while this proportion is lower among adult and elderly women (67.5% and 64.5% respectively). The percentage of women that report emotional IPV is greater among elderly women (66%) and adult women (63%) than young women (51%).

Independently of the rest of the variables included in the model (Table 2), being an immigrant increases the probability of experiencing physical and/or sexual IPV in adult women (PR: 1.91) and the probability of experiencing psychological IPV in young women (PR: 1.51) y adult women (PR: 1.61). Having a higher education level decreases the probability of experiencing physical/sexual IPV in all stages of life (p_young_ <0.001, p_adults_: 0.024 p_elderly_: 0.004) as well as the probability of experiencing psychological IPV in young women (p: 0.011). Using actively employed women as the reference group, unemployment increases the likelihood of experiencing both types of IPV, an association that is statistically significant in adult women (physical/sexual-IPV, PR: 1.7; psychological-IPV, PR: 1.3).

**Table 2:** Prevalence and variables associated with the probability of experiencing IPV in the past 12 months, by age group. Spanish Macrosurvey on Violence Against Women, 2014.

**Physical/sexual violence by a partner or ex-partner in the past 12 months vs. no IPV.**												
** **	**Young people**				**Adults**				**Elderly**			
	**16–29 years**				**30–49 years**				**Mayor de 50 years**			
	**Prev**^**a**^**(%)**	**PR**^**b**^	**[CI 95% ]**^**c**^		**Prev(%)**	**PR**	**[CI 95% ]**		**Prev(%)**	**PR**	**[CI 95% ]**	
Age (years)		0.92	0.85	0.99		1.00	0.97	1.03		0.96	0.93	0.99
Country of birth												
Spain	5.9	1.00			3.3	1.00			2.2	1.00		
Foreigner	7.1	0.95	0.57	1.59	7.1	1.91	1.29	2.85	4.8	1.98	0.87	4.50
Education level												
Up to primary	18.9	1.00			8.8	1.00			2.9	1.00		
Secondary	5.7	0.34	0.21	0.55	4.3	0.67	0.41	1.10	2.2	0.51	0.29	0.90
Higher ed.	2.1	0.16	0.06	0.44	2.2	0.41	0.22	0.78	1.0	0.20	0.06	0.63
Employment situation												
Working	4.6	1.00			3.2	1.00			2.5	1.00		
Unemployed	8.9	1.34	0.78	2.33	6.3	1.71	1.18	2.47	3.1	1.08	0.51	2.29
Other	5.0	0.67	0.32	1.41	3.0	0.81	0.39	1.68	2.2	1.24	0.65	2.37
Are there people who are concerned about you and your wellbeing?												
Always/almost always	5.9	1.00			3.3	1.00			1.9	1.00		
Never/almost never/sometimes.	11.3	1.48	0.75	2.93	11.8	2.72	1.77	4.18	6.1	2.82	1.63	4.90
Physical and/or sexual violence in childhood												
No	4.5	1.00			3.6	1.00			2.1	1.00		
Yes	17.2	3.44	2.19	5.40	8.1	2.14	1.32	3.48	7.0	3.08	1.58	6.01
**Psychological violence only by a partner or ex-partner in the past 12 months vs. no IPV.**												
** **	**Prev(%)**	**PR**	**[95% CI]**		**Prev(%)**	**PR**	**[95% CI]**		**Prev(%)**	**PR**	**[95% CI]**	
Age (years)		0.96	0.92	1.00		1.00	0.98	1.01		1.01	0.99	1.02
Country of birth												
Spain	16.2	1			10.4	1			11	1		
Foreigner	25.9	1.51	1.16	1.95	18.5	1.61	1.31	1.98	13.6	1.32	0.87	2.00
Education level												
Up to primary	29.1	1.00			15.8	1.00			12.2	1.00		
Secondary	18.6	0.70	0.51	0.95	12.0	0.80	0.58	1.11	10.8	0.92	0.74	1.16
Higher ed.	10.5	0.48	0.28	0.80	10.6	0.72	0.50	1.03	8.6	0.69	0.48	0.98
Employment situation												
Working	16.7	1.00			11.2	1.00			10.2	1.00		
Unemployed	16.1	0.83	0.60	1.13	14.7	1.26	1.03	1.53	12.3	1.06	0.76	1.47
Other	20.5	0.93	0.68	1.27	8.0	0.66	0.44	1.01	11.3	1.01	0.75	1.36
Are there people who are concerned about you and your wellbeing?												
Always/almost always	17.5	1.00			11.0	1.00			10.1	1.00		
Never/almost never/sometimes	24.7	1.39	0.92	2.10	19.9	1.51	1.17	1.94	18.8	1.68	1.32	2.14
Physical and/or sexual violence in childhood												
No	16.2	1.00			10.2	1.00			9.9	1.00		
Yes	29.8	1.71	1.31	2.24	26.5	2.41	1.96	2.98	27.2	2.66	2.07	3.41

^a^ Prevalence

^b^ Prevalence ratio

^c^ Confidence Interval 95%

The lack of social support increases the likelihood of both types of IPV in adult women and elderly women. This variable shows a greater magnitude of association with physical and/or sexual violence among both age groups (PRadults: 2.7; PRelderly: 2.8).

The exposure to physical and/or sexual abuse in childhood increases the probability that a woman suffers from current physical and/or sexual IPV and psychological IPV only. This association remains across all of the life stages, increasing the risk that a young woman will experience physical/sexual IPV by 3.4 times and an increase by 2.7 times the risk of psychological IPV for adult women.

Table 3 shows the association between women’s state of health and exposure to IPV in the past 12 months. These association measures are independent of age, place of origin, having a partner, education level, employment situation, social support and exposure to physical and/or sexual violence in childhood.

**Table 3:** Association between women’s state of health and exposure to different types of intimate partner violence in the last 12 months. Spanish Macrosurvey on Violence Against Women, 2014.

Type of intimate partner violence, last 12 months	STATE OF HEALTH VARIABLES, past 12 months										
	Young people				Adults				Elderly		
	16–29 years				30–49 years				Over age 50		
	PR^a^	[95% CI]			PR^a^	[95% CI]			PR^a^	[95% CI]	
	**PERCEIVED HEALTH: State of perceived health, last 12 months, regular/poor /very poor.**										
Physical and/or sexual violence by a partner/ ex-partner (ref: no IPV)	2.59	1.74	3.87		1.68	1.36	2.09		1.28	1.06	1.53
Psychological violence only by a partner /ex-partner (ref: no IPV)	1.35	0.98	1.09		1.37	1.17	1.61		1.19	1.08	1.30
	**MENTAL HEALTH**^**b**^**: People with four or more symptoms, experienced frequently in the past year related to mental health.**										
Physical and/or sexual violence by a partner/ ex-partner (ref: no IPV)	3.1	2.25	4.29		2.61	2.12	3.21		2.17	1.75	2.70
Psychological violence only by a partner /ex-partner (ref: no IPV)	2.24	1.65	3.07		2.16	1.84	2.53		1.69	1.46	1.96
	**LIMITATIONS: People with limitations in daily activities that has lasted or is foreseen to last more than one year.**										
Physical and/or sexual violence by a partner/ ex-partner (ref: no IPV)	2.44	1.41	4.23		1.98	1.47	2.67		1.24	0.92	1.66
Psychological violence only by a partner /ex-partner (ref: no IPV)	1.44	0.98	2.12		1.30	1.13	1.63		1.18	1.02	1.35

^a^ Prevalence ratio adjusted by age, place of origin, having a partner, education level, employment situation, social support, exposure to physical violence and/or sexual violence in childhood.

^b^ Refers to a list of eight symptoms that include: crying for no reason, changes in moods, anxiety or anguish, lack of sex drive, irritability, insomnia, permanent fatigue, sadness and feelings of worthlessness.

Among all of the age groups analyzed, women exposed to physical and/or sexual IPV in the past 12 months have a greater probability of having poor self-perceived health (PRyoung: 2.59; PRadults 1.68; PRelderly:1.28), and a greater probability of having poor mental health (PRyoung: 3.10; PRadults 2.61; PRelderly:2.17). In the case of young and adult women, exposure to current physical and/or sexual IPV is associated with a greater level of activity limitations in daily life (PRyoung: 2.44; PRadults:1.98)

Exposure to only psychological IPV increases the likelihood of having poor self-perceived health in adult and elderly women (PRadults 1.37; PRelderly 1.19). Exposure to psychological violence increases the likelihood of heaving poor mental health in all age groups (PRyoung: 2.24; PRadults 2.16; PRelderly:1.69), as well as limitations in daily activities among adult women (PR: 1.30) and elderly women (PR: 1.18)

## Discussion

The prevalence of physical and/or sexual IPV in the last 12 months is greater among young women than among the rest of the age groups analyzed, but it is of less severity. Among the factors that independently increase the probability of experiencing IPV, a history of physical/sexual violence in childhood is the variable with the greatest magnitude of association for all of the groups analyzed.

Being an immigrant increases the probability of all types of IPV in the adult population and the probability of psychological IPV among young women. Some factors, however, are protective factors, for example having a higher level of education is related to a lower probability of experiencing physical and/or sexual violence among young women. In adult women being unemployed and having low social support increases the likelihood of experiencing both physical/sexual IPV and psychological IPV. The results confirm the negative effects on health of any type of IPV on all of the groups analyzed. The magnitude of the impact of violence on health is greater in women of younger ages.

Young women are a group that is especially at risk for all of the types of violence and especially psychological violence. The prevalence of IPV identified in our work on young women coincides the prevalence shown in the European Union, where 6.1% of ever-partnered young women have been exposed to current physical and/or sexual IPV [5] and 29% of young women have been exposed to psychological IPV only in their lifetime. [8] The lower IPV prevalence shown in adult women compared to young women could be the result of older women’s greater capacity to put to use strategies to exit from violent partner relationships. In Spain, 52% of women ages 35–54 who were exposed to IPV filed a police report, compared to 24.2% of young and elderly women. [18] The decrease in the prevalence of IPV among elderly women, compared to young and adult women, could be related to the presence of other variables associated with IPV, such as the lower presence of minors in the home, or a lower proportion of elderly among the immigrant population. [19] We also cannot discard that women’s perception of IPV changes with age. [20,21] The different type of education received, the greater economic dependence and less functional capacity could serve to normalize situations of IPV, which in other stages of life wouldn’t have been acceptable. In this sense, 9.4% of Spanish women over age 65 consider that IPV is something that has always existed, compared to 3.5% of the population ages 18–24. [22] However, in the group of women who are adults and elderly, other more severe forms of physical violence are more prevalent, as observed previously. [15] It could be that in these elderly women, there are cases of longer duration violence and/or revictimization, which could be responsible for the increase in severity observed. [23]

Severe physical violence affects nearly one in two young women exposed to physical violence. The fact that femicides occur principally among adult women, [22] as well as the higher prevalence of psychological violence related to control among young women could generate a false perception that the IPV suffered by young women is less severe. [24] This is worrisome, given that the perceived severity of an IPV situation, both at the individual and social levels, is a factor related to the search for help [9] and thus putting protective measures in place.

A history of violence in childhood, immigrant status and lower level of education are clearly adverse social circumstances that increase the probability of IPV in any of the age groups studied. [16] The exposure to physical and/or sexual abuse in childhood is the variable that presents the greatest magnitude of association with IPV among all of the age groups analyzed. The impact of physical and/or sexual violence in childhood remains throughout life and leads to a three-fold increase in the probability of experiencing physical and/or sexual IPV in young and elderly women and a two-fold increase in the probability of experiencing psychological violence. The prevention of abuse in childhood is a fundamental pillar in addressing IPV, [25] given the impact of this violence in present and future wellbeing of women. [26–28]

Immigrant status seems to be associated with a greater probability of experiencing any type of IPV in the adult population and psychological IPV in among young women. In Spain immigration is mostly economic in nature, and immigrants are of working ages and come from different regions in Latin America, Africa and Asia, where there is greater gender inequality [29] and IPV prevalence than in our region. [5,7] It is possible that these gender inequalities, and therefore IPV, are reduced among young immigrants due to the influence of the host country. These gender inequalities could be maintained in the adult immigrant population due to a lower cultural permeability of the adult population in the host society [30] It is also possible that immigrant women have more difficulties escaping situations of violence than native-born women, as shown in prior studies. [10]

The fact that adult women who are unemployed have a greater probability of experiencing all of the types of IPV analyzed when compared to employed women could be explained by the difficulty these women have escaping violent relationships when they are economically dependent. [31] In this sense, policies for labor integration of women who experience inter-partner violence could play an important role in the process of their recovery.

The negative impact of IPV on mental health, physical health and daily activity of women has been shown broadly in prior studies. [4,32–35] This study shows that these consequences of IPV can also occur at any time in the lives of affected women, including in young and adolescent women, from their first abusive relationships and more than doubling the probability of chronic limitation in daily activity in addition to poor mental health. Therefore, the response of the health and social systems have a key role in mitigating the negative effect of IPV on health. Studies recently published in Spain show that in recent years, the austerity measures put into place in response to the 2008 economic crisis negatively affected the ability of primary healthcare professionals to detect and respond to women exposed to IPV. [36]

Our results should be interpreted in light of several limitations. On one hand, the cross-sectional study design does not allow us to identify causal relationships, given that we cannot identify the time periods between cause and effect. In any case, given that our result variable refers to the current time, this minimizes the possibility of erroneous classification that can occur with a broader time period for variables at the study level. It is important to signal that this survey at no time defines what a partner relationship is. It is possible that the concept of partner changes among the different social groups studied and that occasional relationships are considered partnerships by some women and not by others. This fact, in addition to the sensitivity of the information collected could cause an under-representation of IPV prevalence.

Despite the limitations described here, it is important to call attention to the magnitude of physical and/or sexual and psychological IPV among younger women, above all taking into account the grave consequences for health observed among this group of women. Putting into place health services and are youth-friendly could serve as an opportunity to improve the response to IPV among women, as has been observed in the cases of health services for sexual and reproductive health. [37]

This study identifies both common and differential factors associated with IPV. Among the common factors, it is important to highlight the vulnerability of women with a history of violence in childhood and those with low education levels at any age. Approaching IPV requires integral strategies that include both addressing school abandonment [8] as well as primary prevention, early detection and treatment of the consequences of violence in childhood. Addressing unemployment and labor integration strategies for adult women could help reduce the prevalence of current IPV in this group of women. Promotion of support networks should be a part of the response aimed at both adult and elderly women. Specific actions are required in the case of adult immigrant women, women that the greater prevalence of violence is independent of the rest of the sociodemographic variables studied. The persistence of these risk factors, some quantitatively and qualitatively different by age suggest the urgent need to link prevention of gender violence to the social circumstances of the population.
